# Cognitive behavior therapy-based psychoeducational groups for adults with ADHD and their significant others (PEGASUS): an open clinical feasibility trial

**DOI:** 10.1007/s12402-014-0141-2

**Published:** 2014-05-27

**Authors:** T. Hirvikoski, E. Waaler, T. Lindström, S. Bölte, J. Jokinen

**Affiliations:** 1 Department of Women’s and Children’s Health, Pediatric Neuropsychiatry Unit, Center for Neurodevelopmental Disorders at Karolinska Institutet (KIND), CAP Research Center, Karolinska Institutet, Gävlegatan 22B, 11330 Stockholm, Sweden; 2 Habilitation and Health, Stockholm County Council, Stockholm, Sweden; 3 Division of Child and Adolescent Psychiatry, Stockholm County Council, Stockholm, Sweden; 4 Department of Clinical Neuroscience/Psychiatry, Karolinska Institutet and Stockholm County Council, Stockholm, Sweden

**Keywords:** ADHD, Family members, Psychoeducation, Treatment, Group, Multimodal

## Abstract

The aim of this pilot study was to investigate the feasibility and effectiveness of a new psychoeducative intervention program (PEGASUS) for adults with ADHD and their significant others in a psychiatric outpatient context. At three outpatient psychiatric clinics, adults with ADHD and their significant others took part in PEGASUS, a psychoeducational program based on theories from cognitive behavioral therapy, neuropsychology, and cross-disciplinary evidence regarding ADHD. In total, 108 adults were allocated to treatment (51 with ADHD and their 57 significant others). Feasibility was evaluated regarding suitability of the intervention at a psychiatric outpatient clinic and treatment completion. Preliminary efficacy was evaluated *per protocol* from baseline to post-intervention (*n* = 41 adults with ADHD and 40 significant others). In a feasibility analysis, the intervention was judged to be a suitable treatment option for 94.5 % of all individuals with a primary diagnosis of ADHD at an outpatient psychiatric clinic. In total, 43 out of 51 allocated individuals with ADHD (84.3 %) completed the intervention. The corresponding figures for their significant others were 42 out of 57 (73.7 %). Knowledge about ADHD increased, and both the quality of relationships and psychological well-being improved from baseline to post-intervention in all participants. The significant others reported a reduction in the subjective burden of care, such as worry and guilt. The objective burden of care (such as financial problems) did not change. The findings support the potential value of psychoeducation for adults with ADHD and their significant others. An ongoing randomized controlled trial will generate further evidence concerning the PEGASUS program.

## Introduction

Attention deficit hyperactivity disorder (ADHD) is an early-onset neurodevelopmental disorder characterized by profound difficulties with inattention, hyperactivity, and impulsivity (American Psychiatric Association 2013). In the majority of cases, ADHD persists into adulthood (Spencer et al. 2007), and the cross-national prevalence rate has been estimated to 3.4 %, assessed in Belgium, Colombia, France, Germany, Italy, Lebanon, Mexico, the Netherlands, Spain, and the USA (Fayyad et al. 2007). Adult ADHD is often accompanied by a heightened susceptibility to various stressors and is associated with pervasive impairments across multiple domains of life, such as mental and physical health, education, work, economy, social life, family living, and parenting (Barkley 2002; Bolte et al. 2013; Brod et al. 2006; Goodman 2007; Hirvikoski et al. 2009). The clinical picture is often further complicated by the presence of additional comorbid psychiatric conditions (Fayyad et al. 2007; McGough et al. 2005; Sobanski et al. 2007).

It has been suggested that the often strained relationships between adults with ADHD and their spouses, family members, friends, and co-workers may result from a combination of mutual, long-term frustration with the symptoms and a lack of understanding of the disorder (Goodman 2007; Moss et al. 2007). Adults with ADHD often struggle with significant difficulties involving emotional regulation (Retz et al. 2012), and their partners and family members often complain about them being forgetful, overreactive, and poor at listening (Murphy 2005). Significant others frequently feel overburdened by the responsibilities of taking care of their family member with ADHD (Cadman et al. 2012; Murphy 2005).

Knowledge pertaining to ADHD is generally low in society at large, and individuals with ADHD, as well as those close to them, are at high risk of being confronted with stigma, prejudices, and discrimination (Mueller et al. 2012). Without an adequate explanation to make sense of their difficulties, individuals with ADHD may perceive ADHD-related misbehaviors as reflecting personal flaws rather than their disorder (Fleischmann and Fleischmann 2012; Young et al. 2008). Before coming to terms with the established ADHD diagnosis, it is not uncommon to encounter emotional turmoil and confusion characterized by negative thoughts and rumination (Young et al. 2008).

Clinical guidelines recommend integrating pharmacological and psychosocial interventions in the treatment of adult ADHD (CADDRA 2008; Ebert et al. 2003; NICE 2009; Practice Parameters 1997). During the last decade, some controlled trials have found cognitive behavioral therapies (CBTs, such as cognitive therapy, dialectical behavioral therapy, and meta-cognitive therapy) to be a promising treatment strategy for adults with ADHD (Emilsson et al. 2011; Hirvikoski et al. 2011; Philipsen et al. 2013; Safren et al. 2010; Solanto et al. 2010; Stevenson et al. 2002). However, CBTs often put high demands on the participants’ motivation, skills, and stamina and thus may not be a suitable option for all individuals in all phases of the care process (Hirvikoski et al. 2011).

Often offered in addition to the standard medical care, psychoeducation is a well-established, evidence-based intervention for several psychiatric disorders in adulthood (Murray-Swank and Dixon 2004). Psychoeducational interventions are aimed at empowering patients and their significant others with knowledge and directly ask patients to share in their own treatment (Hayes and Gantt 1992). While the efficacy of psychoeducational family programs targeting children and adolescents with ADHD has gained support (Montoya et al. 2011), research into psychoeducation for adults with ADHD is still surprisingly scarce. Moreover, the only study published on psychoeducation for adults with ADHD did not involve significant others and resulted in multifaceted findings, indicating positive effects on, for example, disorganization, inattention, and emotional liability, but also potentially negative effects on self-esteem (Wiggins et al. 1999).

We are currently evaluating a manualized psychoeducational program (PEGASUS) (Hirvikoski et al. 2013a) designed as an initial nonpharmacological treatment option after receiving an ADHD diagnosis in adulthood. The aim of the present open clinical trial was to do a pilot study on the feasibility and preliminary effectiveness of the PEGASUS program in a psychiatric outpatient setting. A further aim was to gather feedback from participants and course group leaders that could be used to improve and fine-tune PEGASUS prior to a randomized controlled study.

## Methods

The intervention was conducted as part of the clinical routine at two outpatient tertiary psychiatric clinics for the assessment and treatment of adults with neurodevelopmental disorders (Neuropsychiatric Unit Karolinska, Psychiatry Northwest, and Neuropsychiatric Unit, Psychiatry Southwest, Stockholm County Council) and one outpatient psychiatric clinic (Liljeholmen Outpatient Psychiatric Clinic, Psychiatry Southwest, Stockholm County Council). The study was approved by the Regional Ethics Committee of Stockholm (2009/824-31/3).

### Participants

Participants were recruited from the patient base of the three psychiatric clinics involved in the study. The ADHD diagnostic assessment was performed before the participant entered the study and was based on clinical practice in Stockholm County Council clinics at the time of the study. Multiple sources of information were combined to constitute a consensus between the clinicians involved in the assessment. A clinical interview based on the DSM-IV-TR criteria (American Psychiatric Association 2000) was conducted, and patients completed standardized self-rating questionnaires, such as the Wender Utah Rating Scale (WURS) (Ward et al. 1993) for the assessment of childhood ADHD symptoms and the Adult ADHD Self-Report Scale (ASRS) (Adler et al. 2006) for the assessment of ADHD symptoms in adulthood. The clinical routine involved collecting collateral information from significant others (possible in 88 % of cases in the present study sample), using clinical interviews and/or questionnaires in order to obtain multiperspective diagnostic information on each individual. When available, additional information was obtained from records from child and adolescent psychiatry units and school health services, as well as adult psychiatry services. In most cases, the assessment also included psychological testing, such as estimations of general cognitive capacity (Wechsler 1997) and urine drug screening.

In order to include a sample reflecting the natural heterogeneity of the adult ADHD population presenting in an outpatient psychiatric context, the inclusion criteria for the study were broad: ADHD as the primary (neurodevelopmental) diagnosis; age of 18 years or older; possibility to participate with at least one adult significant other. The exclusion criteria were as follows: current substance abuse (during the previous 3 months); mental retardation (IQ ≤ 70); organic brain injury; autism spectrum disorder; suicidality; any other severe psychiatric disorders (e.g., psychosis); or adverse psychosocial circumstances (e.g., being homeless), thus making successful participation unlikely or impossible. Ongoing pharmacological treatment was not a reason for exclusion.

### Recruitment process and enrollment of participants

The first contact with the ADHD participants was established by sending out study information letters. Thereafter, they were invited to visit the clinic in small groups for further information and for judging the inclusion criteria individually. All participants gave their written informed consent before completing the questionnaires. An experienced clinician (the course coordinator or a professional under the supervision of the course coordinator) conducted individual interviews and studied case files in order to further assess eligibility. Participants with ADHD were instructed to participate with at least one significant other with whom they had a relationship in their everyday lives. The significant others completed the questionnaires at home after having received a written rationale and instructions.

### Psychoeducational program

The PEGASUS program for adults with ADHD and their significant others is a highly structured manualized psychoeducational intervention designed to constitute a first nonpharmacological intervention after the establishment of an ADHD diagnosis at adult age (Hirvikoski et al. 2013a).

The overarching goal of the treatment is to increase the participant’s knowledge of ADHD that may facilitate the management of ADHD in daily life. The information covered not only knowledge of ADHD as such but also different strategies, treatments, and support options provided by psychiatric care and other organizations in society. Further goals are to improve the quality of the relationship between the co-participants (i.e., between the individuals with ADHD and their participating significant other(s)) in order to reduce the burden of care on the participating significant others, to increase acceptance of the ADHD diagnosis, to promote belief in finding relief, and to improve the quality of life of the participants.

The program is based on general principles taken from CBT, neuropsychology, and cross-disciplinary clinical evidence pertaining to ADHD. Several experienced psychologists, psychiatrists, and occupational therapist have contributed to the contents of the program. Following training in the general principles of the intervention, the group leaders were responsible for the staging of the course at the participating clinics. The group leaders were provided with a preliminary version of the workbook (Hirvikoski et al. 2013a), as well as with all materials needed for the implementation of the intervention, such as lecture materials, instructions for lecturers at different sites, informative material for the recruitment.

The PEGASUS program comprises eight sessions in a closed group, including both participants with ADHD and their significant others (Table 1). Each session lasts for 2.5 h and includes a 30-minute break (with coffee/tea and sandwiches). The group leaders accompany the group from the recruitment to the follow-up measures and serve as contact persons for both participants and lecturers. Different lecturers are recruited from the local clinic by the group leader. The lecturers, all with long-standing experience of and expertise in the different course themes (Table 1), are provided with preprepared lecture materials, including power point and planned themes for small group discussions that are organized during the lectures.

**Table 1:** Themes and main focuses

Themes and main focuses of the eight course evenings	The lecturer recruited by the course group leader
*1. Introduction to ADHD in adulthood:* Gives the participants a joint, basic understanding of the ADHD diagnosis, as well as of common difficulties (including psychiatric comorbidity) and strengths for individuals with ADHD	The first lecture should preferably be given by the senior course group leader
*2. Pharmacological and psychological treatment*: Introduces and describes available treatment strategies and options	Psychiatrist and psychologist experienced in the treatment of ADHD in adults
*3. Lifestyle factors: sleep, stress, diet, and exercise*: Focuses on the connection between general lifestyle factors (such as sleep and physical activity) and ADHD symptom severity	Psychologist, occupational therapist, nurse, or other professional experienced in the theme of the lecture
*4. Structure and strategies in everyday life*: Presents a range of strategies and cognitive aids developed to ease life of individuals struggling with executive difficulties	Occupational therapist experienced in ADHD in adults
*5. Living with ADHD*—*acceptance and change*: Focuses on life with ADHD, as experienced and related by an individual having received the diagnosis as an adult^a^	An individual with an ADHD diagnosis
*6. ADHD in relationships*: Focuses on how ADHD symptoms, such as inattention and impulsivity, may affect social behaviors and close relationships. Both positive and negative aspects of ADHD in relationships are discussed from the perspectives of adults with ADHD and significant others	Psychologist, social worker or other professional experienced in the theme of the lecture
*7. ADHD at work*: Informs about the various support measures provided by the employment services and on how job assignments/the workplace may be adjusted based on ADHD symptoms	Guest lecturer(s) from local employment services and psychologist, occupational therapist or other professional experienced in ADHD in the workplace
*8. Service and support provided by society*: Informs about the various support measures society may provide individuals with ADHD^a^	Guest lecturer from local municipality services, social worker or other experienced professional

^a^A representative from the interest organization Attention informs briefly about their work in conjunction with course session 5 or 8

The lecturers are informed both orally and in writing about the general goals and principles of the intervention. The group leader actively strives to support the lecturer so as to keep the intervention in line with the intended general principles. Thus, the focus on psychoeducation (not family counseling, psychotherapy, or individual problem solving) is stressed. Negative experiences, such as school failure or past substance abuse, should be validated while rumination and dwelling on the past are avoided (Young et al. 2008). The preprepared lecture material and the course coordinator support the lecturers in giving their lectures in an empowering and validating spirit, discussing difficulties and disabilities, but also highlighting possibilities for change as well as pointing out common strengths in individuals with ADHD, thus applying techniques of acceptance. The lecturers are also provided with various pedagogical tips to help them give the lectures in an ADHD-friendly way, e.g., facilitating sustained attention and learning process in different ways.

At the time of the present study, the workbook for participants (Hirvikoski et al. 2013b) was not yet published. Instead, all participants received a folder to collect and organize information and handouts. The folder served as a workbook and compendium to make the course material available at home between the sessions and after completing the course.

### Group sizes

The PEGASUS intervention is designed to be carried out in relatively large psychoeducational groups. The group sizes in the present study ranged between 20 and 30 individuals, approximately half of each group consisting of adults with ADHD and half of their significant others. In total, four psychoeducational groups were conducted.

## Measures

### Background and demographic data

Case histories and socio-demographic data on participants with ADHD were extracted from their clinical files. Moreover, they completed a questionnaire covering demographic information and current stressors in different areas of life (Hirvikoski et al. 2009). A modified version of this questionnaire was used to assess the background and demographic data of the significant others.

### Outcome measures

In the present open pilot study, the main assessments regarded feasibility. Moreover, efficacy-related measures were included for a preliminary estimation of treatment effects. Self-rating questionnaires were distributed at baseline of 1–2 weeks before the intervention started (T1), at post-treatment of 1–2 weeks after the last session (T2), and at follow-up of 6 months after the intervention had ended (T3).

### Feasibility

Two criteria were used to evaluate feasibility: (1) the psychoeducative program should be regarded as a suitable intervention for at least 90 % of all individuals assessed with ADHD, as judged in a consecutive cohort from one of the participating clinics by a senior clinical psychologist involved in the project (EW); and (2) a dropout rate of <25 % (i.e., a clear majority should complete the program and thus attend at least 50 % of the sessions). *Treatment satisfaction* was evaluated for the entire psychoeducational program, using a modified version of the patient evaluation form (Hesslinger et al. 2002, 2004, 2010; Hirvikoski et al. 2011; Philipsen et al. 2007), scored on a Likert scale ranging from 0 (“I disagree”) to 4 (“I strongly agree”), and completed anonymously at the end of the last session. The participants also rated the course as a whole following the school grading system “Failed,” “Passed,” “Passed with distinction,” and “Passed with special distinction” (scored 0–3 in the database). To get feedback on each course session for further development of the program, the participants also completed the session evaluation form (SEF) (Bramham et al. 2009), modified for the current study. The SEF was completed anonymously at the end of each course session.

### Efficacy-related measures

All participants completed the ADHD 20 Questions, a knowledge quiz with 20 true/false scored items, reflecting knowledge about ADHD and modified for this study from a corresponding scale (Bramham et al. 2009). Furthermore, all participants completed the questions about family members (QAFM) (Hansson and Jarbin 1997). The QAFM is a dyadic self-report questionnaire (completed with respect to each relationship if the adult with ADHD participated with more than one significant other) that was used to measure aspects of the quality of the relationship between the co-participants (that is, between the adult individual with ADHD and his/her significant other[s]). The QAFM comprises four subscales (Hansson and Jarbin 1997): (1) critical remarks (critical remarks directed at the other person); (2) (the respondent’s) emotional overinvolvement; (3) perceived criticism from the other person; and (4) perceived emotional involvement from the other person in the relationship. The thirty items are scored on a 5-point Likert scale from 1 (“almost never”) to 5 (“almost always”). Low scores on the first three subscales are indicative of a good quality of relationship, while on the last subscale (Emotional Involvement), high scores indicate the same. Symptoms of depression were measured using the Beck Depression Inventory (BDI) Beck et al. (1961, 1988b), symptoms of anxiety using the Beck Anxiety Inventory (BAI) (Beck et al. 1988a), and subjective stress using the Swedish version of the Perceived Stress Scale (PSS) (Cohen et al. 1983; Eskin and Parr 1996).

*In participants with ADHD,* self-esteem was investigated using Rosenberg’s Self-Esteem (RSE) Scale (Rosenberg 1965), and quality of life using the Adult Attention Deficit/Hyperactivity Disorder Quality-of-Life (AAQoL) Scale (Brod et al. 2006). The twenty-nine items of the AAQoL are scored from 1 (“Not at all/Never”) to 5 (“Extremely/Very often”) and summarized to give an overall score.

*In significant others,* the burden of care was assessed using the Burden Assessment Scale (BAS) (Reinhard et al. 1994), which has two subscales (1) Subjective Burden, such as caregiver’s emotional responses, and (2) Objective Burden, such as financial problems. The scale consists of 19 items scored on a 4-point Likert scale from 1 (“Not at all”) to 4 (“A lot”).

## Statistical analysis

Most continuous scales used to assess outcome were normally distributed. However, the BDI and the BAI showed positively skewed distributions due to many low scores (especially among significant others). The results were similar using nonparametric versus parametric statistical methods and, for the sake of brevity, we chose to report results from the parametric methods only. Outliers were screened for using boxplots. One of the three clinics/sites was not able to perform the T3 assessments due to changes in staffing. In conjunction with individuals not reached for T3 measurements at the other sites, the T3 data were missing for one third of the study groups. Therefore, the data were only analyzed from T1 (baseline) to T2 (post-intervention). For cases (*n* = 6 out of 81, 7.4 %) missing T2 but having T3 (follow-up at 6 months), we imputed the T3 score instead of the missing T2. Thus, the main statistical analyses were performed on all participants who (1) completed the treatment, i.e., were present at at least 4 out of 8 sessions and (2) had T2 or T3 data. Among these individuals (*n* = 81), baseline data were missing for four cases on the ADHD 20 Questions and one case on the PSS. The treatment mean imputation (TMI) (Crowe et al. 2010) was used to replace these data. The efficacy-related measures were analyzed using a series of repeated measures ANOVAs (rmANOVAs), with a baseline score (T1), and post-intervention score (T2, if missing, imputed with the T3 score) as a within-subjects repeated measure factor, and group (ADHD versus significant other) as a between-subjects factor. In this way, we also analyzed whether or not the two groups responded differently to the intervention, as would be indicated by group-by-time interaction effects. When indicated by the Mauchly’s test of sphericity, the rmANOVAs were corrected for violence against an assumption of sphericity using the Huyn-Feldt correction. The effect size was expressed as partial eta squared (*η* ^*2*^) for efficacy-related measures and was interpreted using the guidelines proposed by Cohen: 0.01 = small effect size, 0.06 = moderate effect size, and 0.14 = large effect size (Cohen 1988). The alpha levels were set at *p* ≤ 0.05 for significance and at *p* ≤ 0.10 for a statistical trend.

## Results

### Background and demographic data

Background and demographic data are described in Table 2 for participants with ADHD and in Table 3 for significant others.

**Table 2:** Characteristics of participants with ADHD (*n* = 41)

Age	*M* = 37.56, SD = 10.43
	Range 20–63
Sex	26 males (63.41 %)
ADHD subtype	ADHD combined: 31 (75.60 %)
	ADHD inattentive: 10 (24.39 %)
Years diagnosed with ADHD	Less than 12 months: 23 (56.10 %)
	<2 years: 6 (14.63 %)
	<3 years: 3 (7.32 %)
	<4 years: 4 (9.76 %)
	<5 years: 2 (4.88 %)
	<6 years: 3 (7.32 %)
Pharmacological treatment of ADHD	*n* = 22 (53.66 %)
Any psychoactive drug	*n* = 31 (75.61 %)
At least one comorbid DSM-IV diagnosis	*n* = 27 (65.85 %)
Employment	Full-time work, studying or parental leave: 24 (58.54 %)
	Part-time work, studying or parental leave: 4 (9.76 %)
	Unemployed: 3 (7.32 %)
	Long-term sick leave or disability pension: 10 (24.39 %)
Education	University: 10 (24.39 %)
	Upper secondary school: 23 (56.10 %)
	Nine-year compulsory school or less: 5 (12.50 %)
	Other: 2 (4.88 %)
Full-scale IQ^a^	*M* = 96.53, SD = 13.91, range: 76–124
WURS-25 score^a^	*M* = 53.06, SD = 17.13
ASRS^a^	*M* = 47.25, SD = 13.26

^a^Data extracted from the previous diagnostic assessment and available for 26 (63.41 %) for full-scale IQ; 33 (80.49 %) for WURS-25 score (Wender Utah Rating Scale), and 28 (68.29 %) for ASRS

**Table 3:** Characteristics of participating significant others (*n* = 40)

Age	*M* = 48.48, SD = 15.00
	Range: 20–73
Sex	16 males (40 %)
Employment	Full-time work or studying: 30 (75 %)
	Part-time work: 2 (5 %)
	Retired: 5 (12.50 %)
	Unemployed: 1 (2.50 %)
	Long-term sick leave or disability pension: 2 (5.00 %)
Education	University: 18 (45 %)
	Upper secondary: 16 (40 %)
	Nine-year compulsory school or less: 5 (12.50 %)
	Other: 1 (2.50 %)
Relation to the participant with ADHD	Partner: 19 (47.50 %)
	Parent: 18 (45 %)
	(Grown-up) child: 2 (5 %)
	Close friend: 1 (2.50 %)
Living in the same household as the participant with ADHD	Yes: 31 (77.50 %)
Involved in the diagnostic assessment of the participants with ADHD	Yes: 30 (75 %)
Member of any ADHD interest organization or advocacy group	Yes: 3 (7.50 %)

### Feasibility

The first criterion for feasibility was judged at the Neuropsychiatric Unit Karolinska, where 144 individuals were diagnosed during the years 2004–2008 with ADHD as their main neurodevelopmental diagnosis. The psychoeducational program was estimated to be a suitable intervention for 136 (94.5 %) of the individuals in this consecutive cohort.

**Fig. 1:**
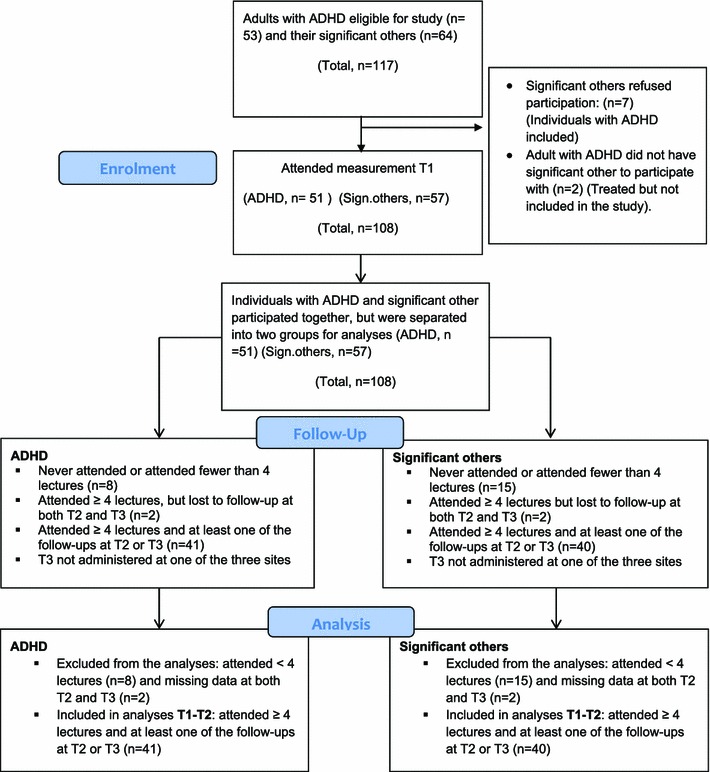
Flowchart for the study group including adults with ADHD and their significant others (SO)

The flowchart for the present study group is presented in Fig. 1. In total, 43 out of the 51 allocated individuals with ADHD (84.3 %) completed the intervention. The corresponding figures for the significant others were 42 out of 57 (73.7 %).

**Fig. 2:**
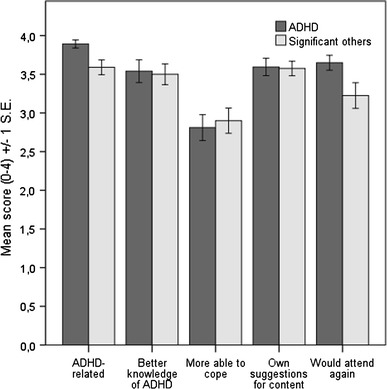
Treatment satisfaction such as measures with patient evaluation form

The mean scores (±1 standard error) on the patient evaluation form are shown in Fig. 2. The overall treatment satisfaction was good among both individuals with ADHD and their significant others. However, the participants with ADHD rated significantly higher than their significant others on the items “The course was clearly related to ADHD” (*p* = 0.007) and “I would attend a similar course in the future” (*p* = 0.03). The rating of the whole course according to the school grading system did not differ between the groups (M = 2.21, SD = 0.72, corresponding to “Passed with distinction”). The feedback on each course occasion was summarized in writing for the purpose of further development of the PEGASUS program.

### Efficacy-related measures

The mean values and standard deviations for questionnaires administered to *all participants* at baseline as well as post-intervention are depicted in Table 4. The results indicated positive improvements in knowledge about ADHD, relationship quality (the QAFM Critical Remarks Subscale), psychological well-being (BDI and BAI), and subjective stress (PSS) over time. The only significant interaction effect was observed in the ADHD knowledge quiz and indicated a somewhat larger increase in knowledge in the significant others from baseline to post-intervention (*F*_(1,79)_ = 19.91, *η* ^*2*^ = 0.06, *p* < 0.05).

**Table 4:** Results of repeated measures ANOVAs from baseline to post-intervention for questionnaires completed by both adults with ADHD and their significant others

Outcome measures	Baseline	Post- intervention	rmANOVA statistics	*p* value
	Mean (SD)	Mean (SD)	*F* (*df*)	*η* ^*2*^effect size
*ADHD*-*20 questions*				
Adults with ADHD	15.76 (2.26)	16.27 (1.82)	*F*_(1,79)_ = 19.91	*p* < 0.001
Significant others	14.60 (2.36)	16.18 (1.84)		*η* ^*2*^ = 0.20
*QAFM perceived criticism*				
ADHD	14.16 (5.37)	14.13 (4.79)	*F*_(1,82)_ = 0.51	ns
Significant others	13.38 (4.63)	12.85 (4.52)		*η* ^*2*^ < 0.01
*QAFM perceived emotional involvement*				
ADHD	14.22 (2.77)	14.69 (3.11)	*F*_(1,82)_ = 0.14	ns
Significant others	12.69 (3.04)	12.49 (2.88)		*η* ^*2*^ < 0.01
*QAFM critical remarks*				
ADHD	20.76 (8.08)	18.84 (6.54)	*F*_(1,80)_ = 11.55	*p* < 0.01
Significant others	23.19 (7.89)	20.95 (6.75)		*η* ^*2*^ = 0.13
*QAFM emotional overinvolvement*				
ADHD	18.51 (6.36)	18.49 (6.21)	*F*_(1,80)_ = 1.04	ns
Significant others	21.68 (6.41)	20.76 (5.46)		*η* ^*2*^ = 0.01
*Beck depression inventory*				
ADHD	18.81 (12.41)	15.39 (10.86)	*F*_(1,79)_ = 8.00	*p* < 0.01
Significant others	9.18 (8.09)	7.65 (7.80)		*η* ^2^ = 0.09
*Beck anxiety inventory*				
ADHD	14.37 (11.43)	12.00 (10.39)	*F*_(1,79)_ = 5.39	*p* < 0.05
Significant others	6.95 (6.72)	6.05 (593)		*η* ^2^ = 0.06
*Perceived stress scale*				
ADHD	32.85 (9.67)	30,39 (9.75)	*F*_(1,78)_ = 4.92	*p* < 0.05
Significant others	23.85 (6.16)	22.08 (8.53)		*η* ^2^ = 0.06

*QAFM* Questions About Family Member questionnaire

In questionnaires completed by the *adults with ADHD*, a trend toward improvement of self-esteem was observed in RSE (*F*_(1,40_) = 3.75, *η* ^*2*^ = 0.09, *p* = 0.06), while the increase in the AaQoL score did not reach statistical significance or trend (*η* ^*2*^ = 0.05, *p* = 0.15).

On the the Burden Assessment Scale (BAS), completed by the *significant others*, a significant decrease was observed in the subjective burden (*p* < 0.01), while no changes occurred in the objective burden from baseline to post-intervention (Table 5).

**Table 5:** Results of repeated measures ANOVAs from baseline to post-intervention for the Burden Assessment Scale completed by significant others only

BAS subscale	Baseline	Post- intervention	rmANOVA statistics	*p* value
	Mean (SD)	Mean (SD)	*F*(*df*)	*η* ^*2*^effect size
Objective burden	0.85 (0.58)	0.84 (0.52)	*F*_(1,39)_ = 0.00	ns
				*η* ^*2*^ < 0.01
Subjective burden	1.08 (0.76)	0.80 (0.55)	*F*_(1,39)_ = 8.73	*p* < 0.01
				*η* ^*2*^ = 0.18

## Discussion

A new manualized psychoeducational program for adults with ADHD and their significant others, PEGASUS (Hirvikoski et al. 2013a), was evaluated in an open study design regarding feasibility and preliminary efficacy. An additional aim was to gather feedback that could be used to inspire and sustain further development of the program before a randomized controlled trial was to be undertaken.

The PEGASUS program was designed to constitute a first psychological intervention after the establishment of an ADHD diagnosis, while the more demanding behavioral therapeutic treatments are planned for later on in an optimized treatment pathway. The psychoeducational program was judged to be a suitable treatment option for 94.5 % of adults with ADHD in an outpatient psychiatric context. Treatment suitability was judged for the adults with ADHD only. In the present study, their significant others were included routinely. Since the PEGASUS program is the first manualized psychological treatment for adults with ADHD that also involves their significant others, one of the challenges was to make the program acceptable and beneficial for all participants—regardless of diagnostic status. The overall treatment satisfaction was good in both groups. However, there were also slight differences between the adults with ADHD and their significant others, namely, adults with ADHD valued the program slightly more in some respects (such as willingness to participate in a similar program in the future). Moreover, treatment completion was better among adults with ADHD (84.3 %) than among their significant others (73.7 %). Therefore, the acceptability of the program for both groups was judged to be one of the main focuses for further development of the PEGASUS program.

One of the main goals of the intervention is to provide the participants with evidence- based knowledge concerning ADHD in order to reduce stigma, prejudices, and discrimination (Mueller et al. 2012) and to increase understanding of the disorder and thereby improve the relationship between the co-participants (i.e., the adult individual with ADHD and his/her participating significant other[s]) (Goodman 2007; Moss et al. 2007). Knowledge pertaining to ADHD was improved from pre- to post-intervention in both groups. Moreover, the measurement of expressed emotions indicated a reduction in critical remarks directed toward the co-participant in the course. In addition, we observed a positive effect on psychological well-being (symptoms of depression, anxiety, and perceived stress) in the entire study group, i.e., both in adults with ADHD and their significant others. The reduced subjective burden on the participating significant others may reflect a better knowledge of ADHD and acceptance of ADHD as a disability.

An important goal of the development and evaluation of all new psychological interventions is to ensure that the intervention is not harmful to the participants. A focus on possible harmful effects has not been a central aspect of research on psychological interventions, although emerging data indicate that several psychological treatments may produce harm in a significant number of individuals (Lilienfeld 2007). Therefore, attention to the well-accepted principle *primum non nocere* (“first, do no harm”) should also be increased among psychologists (*ibid*). This may be especially true regarding psychological treatments for adults with ADHD, since early studies (Ratey et al. 1992) on clinical characterization of adult ADHD indicate that traditional psychological treatment “had little beneficial effects and aggravated problems of self-esteem.”. Indeed, the only study published hitherto on a diagnosis-specific psychoeducational program for adults with ADHD (Wiggins et al. 1999) showed a negative effect on self-esteem. The authors speculated that the decrease in self-esteem may be a temporary effect of increased awareness of the difficulties and problems in everyday life, as well as an understanding of the effort that is needed to manage everyday life while having ADHD. On the contrary, our goal in the PEGASUS program was to increase awareness of problems, as well as the needed coping strategies, while preserving self-esteem. Thus, we used techniques from contextual behavior therapies to promote both coping/change and acceptance in a dialectical, constructive manner. We measured potential effects on self-esteem using the same questionnaire as Wiggins et al. and did not observe any significant effect of the PEGASUS program on self-esteem (i.e., the increase in self-esteem approached, but did not reach, statistical significance). In the further development of the program, the issue of self-esteem has been focused on continuously. As pointed out in the first study focusing on psychological treatments for adults with ADHD, “the critical point for practitioners is that the requirements of treatment for patients with attentional deficits go far beyond just simple treatment of the neurological problem. One needs to consider the ramifications of the disorder in all aspects of the patient’s life: vocational, educational, social, and psychological.” (Ratey et al. 1992). A crucial goal for the PEGASUS program is to provide the participants with the same information, in a way that does not cause harm but hopefully strengthens the participant’s self-esteem and, in the long run, quality of life.

Due to the open study design, the results from the efficacy-related measures should be considered to be preliminary and interpreted cautiously. In an open study, the conventional alpha level of *p* < 0.05 may be considered as rather lenient since the observed effects may partly be related to regression toward the mean at T2/T3. Thus, the randomized controlled study currently under progress will provide more information on the efficacy of the treatment program. Additional limitations were the amount of missing data, especially at the scheduled follow-up of 6 months after the program, and therefore missing long-term follow-up data. Moreover, we observed possible ceiling effects (the knowledge quiz) and floor effects (measures of depression and anxiety among significant others) on some of the outcome measures. Bearing these limitations in mind, the overall pattern in the results indicated promising effects of participation in the PEGASUS program and encouraged us to continue with the project and initiate a randomized controlled trial. However, before entering the RCT phase, the treatment materials were subjected to a thorough adaptation based on feedback from the participants as well as the involved course coordinators.

In summary, a new manualized psychoeducational program for adults with ADHD and their significant others, PEGASUS (Hirvikoski et al. 2013a), was evaluated in an open study design. The results regarding feasibility, treatment satisfaction, and preliminary efficacy were promising.
